# Preclinical research in paclitaxel-induced neuropathic pain: a systematic review

**DOI:** 10.3389/fvets.2023.1264668

**Published:** 2023-12-18

**Authors:** Carolina Bacalhau, José Tiago Costa-Pereira, Isaura Tavares

**Affiliations:** ^1^Department of Biomedicine, Unit of Experimental Biology, Faculty of Medicine, University of Porto, Porto, Portugal; ^2^I3S-Institute of Investigation and Innovation in Health, University of Porto, Porto, Portugal; ^3^Faculty of Nutrition and Food Sciences, University of Porto, Porto, Portugal

**Keywords:** chemotherapy, neuropathic pain, animal models, reproducibility, clinical translation

## Abstract

**Introduction:**

Chemotherapy-induced peripheral neuropathy (CIPN) is a common consequence of cancer treatment and pain is a frequent complaint of the patients. Paclitaxel, a cytostatic drug, generates a well-described peripheral nerve injury and neuroinflammation, which may be experimentally mimicked in animal models. We conducted a systematic review analyzing the experimental design, reporting and mechanisms underlying paclitaxel-induced neuropathy in the included studies to establish the perspectives of translation of the current literature in models of CIPN.

**Methods:**

We elected studies published in Pubmed and Scopus between 1 January 2018 and 3 December 2022.

**Results:**

According to a defined mesh of keywords searched, and after applying exclusion and inclusion criteria, 70 original studies were included and analyzed in detail. Most studies used male Sprague-Dawley rats to induce paclitaxel-induced neuropathy, used low doses of paclitaxel, and the analyzed studies mainly focused at 14-28 days of CIPN. Mechanical nociceptive tests were preferred in the behavioral evaluation. The mechanisms under study were mainly neuroinflammation of peripheral nerves. The overall methodological quality was considered moderate, and the risk of bias was unclear.

**Discussion:**

Despite the ample preclinical research in paclitaxel-induced neuropathy, this systematic review alerts to some flaws in the experimental design along with limitations in reporting, e.g., lack of representation of both sexes in experimental work and the lack of reporting of the ARRIVE guidelines. This may limit the reproducibility of preclinical studies in CIPN. In addition, the clinical features of CIPN should be considered when designing animal experiments, such as sex and age of the CIPN patients. In this way the experimental studies aiming to establish the mechanisms of CIPN may allow the development of new drugs to treat CIPN and translation in the research of CIPN could be improved.

## 1 Introduction

Cancer treatment has a huge impact on patients’ lives. The most common cancer treatment, chemotherapy, typically results in side effects including neuropathy (1). Chemotherapy-induced peripheral neuropathy (CIPN) develops throughout various stages of cancer treatment and may determine a decrease of chemotherapy doses or the interruption of the chemotherapy treatments (2). CIPN may continue even after cancer remission, with a huge impact on the quality of life of cancer survivors (3). The incidence of CIPN may be affected by the chemotherapy protocol, namely cytostatic agent (type and dose) and method of CIPN assessing after cessation of chemotherapy (4). Patients with CIPN commonly express a variety of sensory dysfunctions, including spontaneous pain, hyperalgesia (increased sensitivity to noxious stimuli), and painful reactions to non-noxious stimuli (allodynia). Hypersensitivity to cold and mechanical stimuli are the main symptoms of CIPN (1, 5, 6). Moreover, negative sensory signs along with non-noxious alterations, such as itch, may also occur (7). One of the most used chemotherapy agents, paclitaxel, frequently used to treat breast, ovarian, and lung cancer, was shown to be one of the cytostatic drugs that more commonly induces CIPN (1, 8). Using validated animal models that mimic chemotherapy cycles, paclitaxel-induced neuropathy has been extensively studied in experimental settings (9–11). These rats exhibit several pain-like behaviors namely mechanical and cold hypersensitivity and spontaneous pain (11, 12). Moreover, rodents treated with paclitaxel exhibited anxiety-like and depression-like behaviors (13), as observed in clinical setting (14).

The mechanisms involved in the pathophysiology of paclitaxel-induced neuropathy have been recently studied and reviewed (8). The effects of paclitaxel on neurons at the periphery and spinal cord levels were described however its supraspinal effects remain to be studied (15–20). Our research group developed pioneer studies regarding the neuroplastic changes that occur in pain modulatory brain centers during paclitaxel-induced neuropathy (21, 22), such as the periaqueductal gray and the hypothalamus (23).

The existing preclinical studies of paclitaxel-induced neuropathy mechanisms are yet to produce clinical translation data. This is not exclusive of this neuropathy type since a “crisis of translation” has been discussed in animal pain studies, which was proposed to account for the lack of new analgesic drugs to manage chronic pain (24, 25). The problems derived from the poor degree to which pain neurobiology in rodents may predict pain neurobiology in humans, along with inaccurate experimental design in animal pain studies, should be openly discussed.

In order to assess the quality of preclinical studies using paclitaxel-induced neuropathy in terms of what they can mimic the clinical problems, along with questions of reproducibility and translatability and to identify the mechanisms of paclitaxel-induced neuropathy that are currently under study, we performed a systematic review of the literature that has been published in the last 5 years (2018–2022). The analysis was performed to evaluate the robustness of animal research in paclitaxel-induced neuropathy which may be useful in the identification of mechanisms involved in CIPN seeking be putative translation perspectives.

## 2 Methods

### 2.1 Literature search

The literature search was performed on 03 December 2022 using two electronic databases: PubMed Central (via PubMed) and Scopus to identify preclinical studies of paclitaxel-induced neuropathic pain in last 5 years. For each database, the following syntax was used: *(((paclitaxel) OR (taxol)) AND ((pain) OR (nociception)) AND ((rat) OR (mice)))* from 1 January 2018 to 03 December 2022. The protocol of this review was not registered prior submission.

### 2.2 Selection criteria

Relevant peer-reviewed articles, published in English language, were included based on the following criteria: (1) original articles; (2) studies with rats and/or mice; (3) rodent model of paclitaxel- or taxol-induced neuropathy; (4) only one therapeutic approach (pharmacological or non-pharmacological) with the respective mechanisms of action, signaling pathways and/or target receptors discussed. The exclusion criteria included (1) non-original articles (reviews, clinical trials, case reports or conference abstracts); (2) publications written in languages other than English; (3) studies with other CIPN animal models; (4) combination of pharmacological and/or non-pharmacological therapies.

### 2.3 Study selection

After removing duplicate publications using EndNote, two researchers (CB and JTCP) assessed the eligibility of the articles based on the title and abstract. The second phase of this process, full-text articles were also selected against the following inclusion criteria: (1) previous criteria; (2) full-text access; (3) rodents having the minimum time of experimental condition (paclitaxel- or taxol-induced neuropathy) ≥ 11 days; (4) perform at least 1 nociceptive test (e.g., von Frey, cold and hot plate, Hargreaves or acetone test) to confirm the painful condition underlying the CIPN. A third investigator (IT) acted as mediator of different opinions between the 2 investigators.

### 2.4 Data extraction

The data extraction was carried out by three investigators (CB, JTCP, and IT). The following data were collected from the included articles and compiled, including the following information: reference of the study; characterization of the animal population (species, strain, sex, age, weight, animal supplier, frequency of paclitaxel treatment, dose, route, control group, injection volume, and time of CIPN). For dose of paclitaxel, three categories were created to present the results, studies that used doses of paclitaxel lower than 2 mg/kg, doses greater than or equal to 2 and less than or equal to 8 mg/kg, and studies that used doses greater than 8 mg/kg. Likewise, three categories were created to present the results of duration of CIPN, studies that used animals with a CIPN duration of less than 14 days, greater than or equal to 14 days and less than or equal to 28 days, and a CIPN duration of greater than 28 days.

In addition to these data, it was also extracted information about the main methods (pharmacology, non-pharmacological approaches, cell culture, biochemistry, histopathology, electrophysiology, biochemistry) and, if applicable, the tissues under analysis.

Regarding the behavioral tests, we extracted information about the type of tests and the sensorial modality evaluated, as well as spontaneous pain. Moreover, we also extracted information about types of tests which assess anxiety and depression, and locomotor activity. We grouped the timepoints in weeks (week 1 to week 8). A heatmap were generated where in white are the timepoints not evaluated, in green the timepoints with a positive response and in red the timepoints where a negative response. Furthermore, in yellow are the timepoints where we found more than one different response to the tests in the same week, and in gray are the timepoints where they evaluated the animals but did not report descriptive statistics.

In addition to these data, we also extracted the information to understand which mechanisms underlying paclitaxel-induced neuropathy are being studied as well as the nervous tissues under analysis (peripheral nerves, spinal cord and brain). A summary of the results was reported. The main mechanisms were grouped in classes involving: (1) neuroinflammation, a complex phenomenon involving the activation of the glial cells and release of inflammatory mediators, such as cytokines and chemokines in the nervous system (26); (2) oxidative stress, the imbalance between the production of reactive oxygen species and the ability to remove them, in this specific case, from the neurons (27); (3) cannabinoids and its receptors; (4) opioids and its receptors; (5) monoamines, such as noradrenaline and serotonin, and its receptors; (6) amino acids, which include glutamate and GABA (Gamma-aminobutyric acid), which are, respectively, excitatory and inhibitory neurotransmitters (GABA); and (7) transient receptor potential vanilloid 1 (TRPV1).

### 2.5 Reporting quality assessment

After extracting the data as described above, the studies were analyzed regarding their quality, namely the implementation of the ARRIVE (Animal Research: Reporting of In Vivo Experiments) guidelines and the risk of bias, as described below.

#### 2.5.1 ARRIVE guidelines implementation

The ARRIVE guidelines are a validated checklist used to improve the quality of reporting of animal research (28), which comprise 10 essential (study design, sample size, inclusion and exclusion criteria, randomization, blinding, outcome measures, statistical methods, experimental animals and procedures, and results) and 11 recommended items (abstract, background, objectives, ethical statement, housing and husbandry, animal care and monitoring, interpretation of results, generalization and translation, protocol registration, data access and declaration of interest). Two investigators (JTCP and IT) evaluated the quality of the included studies using the ARRIVE guidelines where each component was classified at three levels of color encoding: red (incomplete) if no sub-item was described, yellow (partly complete) if one or more of the sub-items were described and green (complete) when all sub-items were fulfilled. Adapted from Fonseca-Rodrigues et al. (29), in which each sub-item/color is classified with different scores, each sub-item of the ARRIVE Essential 10 were scored as 1.1, 0.5, or 0 to green, yellow, or red, respectively, and the total score were calculated for each study. If the total score of each study is more than 15, the global rating is considered high, if the score is between 10 and 15, the global rating is considered moderate, and if the score is less than 10, it is considered low. The same scoring method was used for ARRIVE Recommend List, where green was scored 1.25, yellow 0.625 and red zero.

#### 2.5.2 Risk of bias analysis

The SYRCLE’s Risk of Bias Tool is an instrument to assess methodological quality of the animal studies (30). This tool covers ten questions which assess the selection bias, performance bias, detection bias, attrition bias, reporting bias and other biases. Each question was scored by color coding, where high risk, low risk and unclear risk of bias were represented by red, green, and yellow, respectively (31). The score was calculated by two investigators (JTCP and IT).

## 3 Results

### 3.1 Data collection

**Figure 1:**
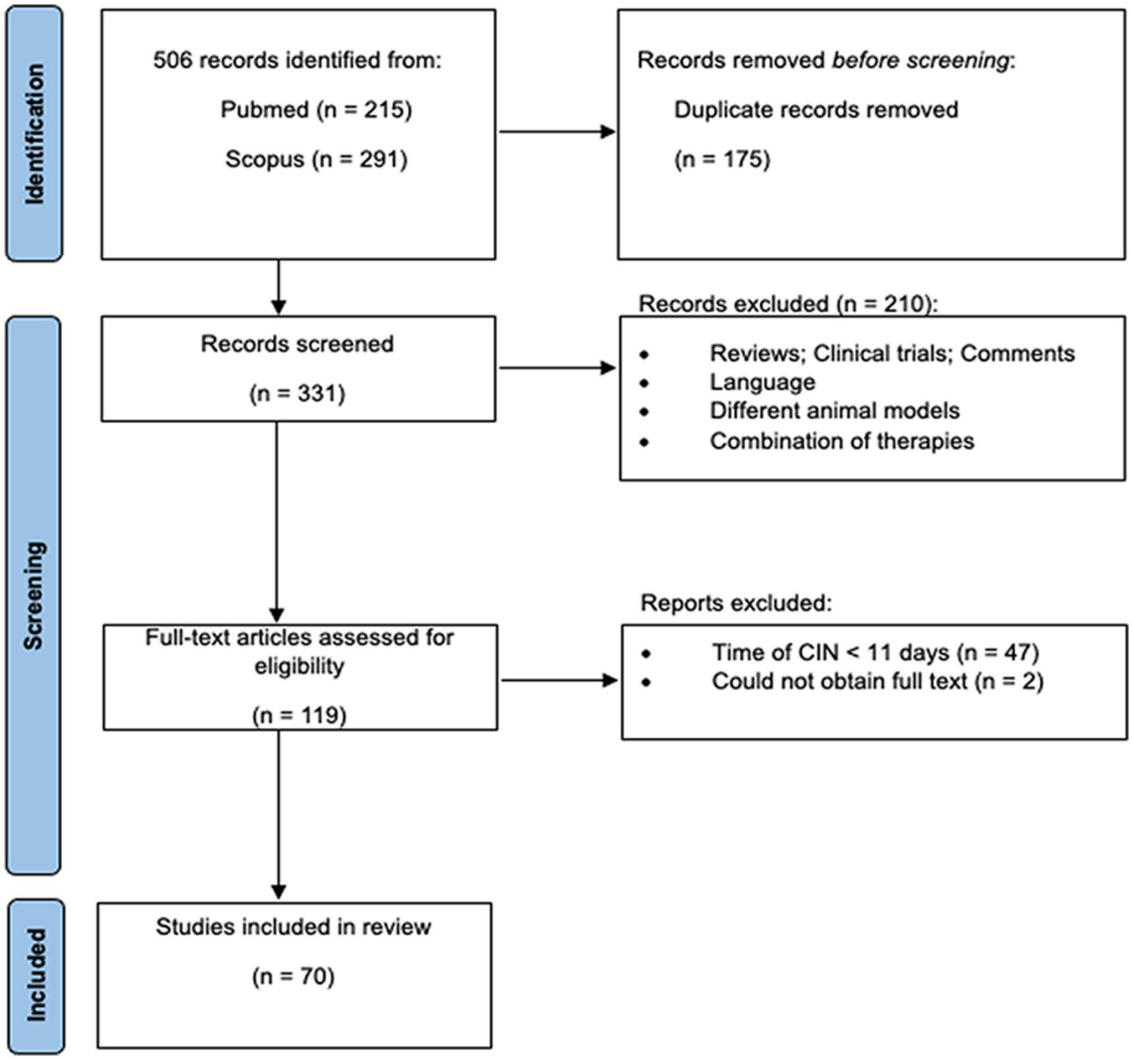
PRISMA flowchart of the different stages of the selection process in the current systematic review.

The PRISMA flowchart of the current study is shown in Figure 1. The electronic search identified 506 studies from PubMed (215) and Scopus (291) as eligible for this systematic review using the search strategy detailed in Section 2. After identifying duplicates, 175 studies were removed, and 331 publications were screened based on the title and abstract. As a result, 210 publications were excluded due to non-compliance with the inclusion criteria. In the second phase, after inspection of the full text of 119 publications, 70 studies were included in this review.

### 3.2 Characterization of the animal population

We analyzed the studies to extract data about animal features, namely species, strain, sex, age, weight, animals’ supplier, total number of animals, number of animals per group and the location of studies.

Rats were the dominant specie studied (41 articles; 58.6%) followed by mice (26 articles; 37.1%) and 3 articles used both rats and mice (4.3%). Regarding the predominant strain, in rats it was Sprague–Dawley (81.8%) and in mice C57BL/6 (55.2%). In some articles using mice the authors used more than one strain (5 articles; 17.2%). One article using rats and one using mice, 2.3% and 3.4% respectively, did not mention any strain in their methods sections (Table 1). In rats, all articles reported the sex of animals. Most articles (88.6%) used male animals, 4.5% and 6.8% used female and both sexes, respectively. In studies using mice, 3 articles (10.3%) failed to mention the animals’ sex. Of the studies that reported, 61.5% used male mice, 34.6% used both sexes and only 3.8% used female mice (Table 1).

**Table 1:** Characterization of the population (species, strain, sex, age, weight, animal supplier, total number of animals, number of animals per group and where were studies performed) in the analyzed studies.

REF	Species	Strain	Sex	Age (weeks)	Weight (grams)	Animal supplier	Total number animals	Number animals per group	Where were studies performed
**Characterization of the population: RATS**									
Li et al. (32)	Rat	Sprague–Dawley	M	-	200–220	BLF (Tongji Medical College)	250	6	Asia
Ma et al. (33)	Rat	Sprague–Dawley	M	8	180–200	BLF (Experimental Animal Center of Hebei Medical University)	ND	6	Asia
Nasser et al. (34)	Rat	Wistar	M	-	170–230	BLF (National Research Center in Cairo)	ND	9	Africa/Asia
Sezer et al. (35)	Rat	Sprague–Dawley	M	6–8	180–200	BLF (Erciyes University Laboratory Animal Care Facility)	42	8	Europe
Wang et al. (36)	Rat	Sprague–Dawley	M	6–8	180–220	CS (Charles River Laboratories)	ND	4 to 6	Asia
Alkislar et al. (37)	Rat	Sprague–Dawley	M	-	300–325	CS (Charles River Laboratories)	ND	6 to 12	America
Chen et al. (38)	Rat	Sprague–Dawley	M	6–7	200–220	BLF (Tongji Medical College)	291	6	Asia
Chou et al. (39)	Rat	Sprague–Dawley	M	7	180–200	CS (BioLASCO Taiwan)	24	6	Asia
Garrido-Suárez et al. (40)	Rat	Sprague–Dawley	M	8–10	200–250	BLF (Center for Experimental Animals Production)	ND	8 to 10	America
Ilari et al. (41)	Rat	Sprague–Dawley	M	8	-	CS (Envigo)	ND	15	Europe and America
Ma et al. (42)	Rat	Sprague–Dawley	M	-	180–200	BLF (The Experimental Animal Center of Hebei Medical University)	ND	6	Asia
Meregalli et al. (43)	Rat	Wistar	F	-	175–200	CS (Envigo Laboratory)	48	12	Europe
Semis et al. (44)	Rat	Sprague–Dawley	M	10–12	285–315	BLF (Experimental Research and Application Center, Ataturk University)	35	7	Europe
Wang et al. (45)	Rat	Sprague–Dawley	M	-	180–240	BLF (Institute of Experimental Animals, Naval Medical University)	ND	6 to 8	Asia
Zhang et al. (46)	Rat	C57BL/6	M	8–10	23–26	BLF (Experimental Animal Center of Peking University)	40	10	Asia
Zhong et al. (47)	Rat	Sprague–Dawley	M	-	120–150	CS (Charles Rivers Labs)	ND	6 to 10	Asia
Brewer et al. (48)	Rat	Sprague–Dawley	M and F	8.57–14.29	-	BLF (In-house colony)	64 M and 63F	4 to 6	America
Costa-Pereira et al. (21)	Rat	Wistar	M	-	175–190	CS (Charles River)	ND	4 to 8	Europe
Costa-Pereira et al. (22)	Rat	Wistar	M	-	190–200	CS (Charles River)	95	5 to 8	Europe
Ferrari et al. (49)	Rat	Sprague–Dawley	M and F	0.29–8	-	CS (Charles River Laboratories)	ND	6 to 12	America
Hacimuftuoglu et al. (50)	Rat	Wistar	F	-	190–210	-	40	8	Europe/Asia
Huang et al. (51)	Rat	Sprague–Dawley	M	-	220–250	BLF (Institute of Experimental Animals of Sun Yat-sen University)	ND	4 to 11	Asia
Kamata et al. (52)	Rat	Wistar	M	-	250–320	-	ND	4 to 10	Asia
Kim et al. (53)	Rat	Sprague–Dawley	M	-	200–350	CS (Harlan Sprague Dawley Company)	ND	8	America
Liu et al. (54)	Rat	Sprague–Dawley	M	-	250–300	-	ND	6	Asia
Wang et al. (55)	Rat	Sprague–Dawley	M	-	200–250	-	ND	6 to 14	Asia
Zhang et al. (56)	Rat	Sprague–Dawley	M	-	220–250	CS (Vital River Laboratory)	ND	12	Asia
Zhao et al. (57)	Rat	Sprague–Dawley	M	-	120–150	BLF (China Academy of Military Science)	29	7 to 8	Asia
Zhou et al. (58)	Rat	Sprague–Dawley	M	-	200–220	BLF (Tongji Medical College)	ND	6	Asia
Zhou et al. (59)	Rat	Sprague–Dawley	M	-	200–220	BLF (Tongji Medical College)	ND	6	Asia
Li et al.	Rat	Sprague–Dawley	M	5–8	180–220	BLF (Shanghai Laboratory Animal Center)	ND	5	Asia
Li et al. (60)	Rat	Sprague–Dawley	M	-	180–200	BLF (Experimental Animal Center of Hebei Medical University)	ND	10	Asia
Sivanesan et al. (61)	Rat	-	M	-	350–400	CS (Envigo)	34	6 to 11	America
Wu et al. (62)	Rat	Sprague–Dawley	M	-	120–150	CS (Charles River Laboratories)	ND	9	America
Wu et al. (63)	Rat	Sprague–Dawley	M	5–6	200–220	BLF (Ningbo University Laboratory Animal Center)	ND	8 to 10	Asia
Al-Mazidi et al. (64)	Rat	Sprague–Dawley	M	-	250–300	-	62	12 to 50	Asia
Ba et al. (65)	Rat	Sprague–Dawley	M	-	200–250	CS (Guangdong province Laboratory Animal Center)	ND	8 to 12	Asia
Legakis et al. (66)	Rat	Sprague–Dawley	M and F	-	360–468 (M)/236–298 (F)	-	39 M and 12F	3 to 6	America
Vitet et al. (67)	Rat	Sprague–Dawley	M	-	250–300	CS (Janvier)	ND	8	Europe
Zhang et al. (68)	Rat	Sprague–Dawley	M	-	250–270	CS (Harlan)	ND	6 to 7	America and Asia
**Characterization of the population: MICE**									
Balkrishna et al. (69)	Mouse	CD-1	M	6–8	20–25	CS (Hylasco Biotechnology Pvt)	ND	6	Asia
Cristiano et al. (70)	Mouse	CD1	M	12	25–30	CS (Charles River Laboratory)	40	8	Europe
Ezaka et al. (71)	Mouse	C57BL/6 J	M	6–7	-	CS (Jackson Laboratory)	ND	8	America
Karmakar et al. (72)	Mouse	Swiss Albino	-	-	25–30	-	25	5	Asia
Lin et al. (73)	Mouse	C57BL/6 J	M and F	-	25–33	BLF (Indiana University)	ND	6 to 11	America
Park et al. (74)	Mouse	ICR	M	6	-	CS (Samtako Bio)	ND	ND	Asia
Paton et al. (75)	Mouse	C57BL/6 J	M and F	8	-	BLF (Victoria University of Wellington (VUW) Animal Facility)	ND	6 to 50	Oceania
Caillaud et al. (76)	Mouse	C57BL/6 J	M and F	12	-	CS (The Jackson Laboratory)	96	8 to 12	America
Caillaud et al. (77)	Mouse	C57BL/6 J	M and F	12	-	CS (The Jackson Laboratory)	ND	8 to 12	America
Cuozzo et al. (78)	Mouse	CD1	M	-	25–30	CS (Charles Rivers)	40	10	Europe
Foss et al. (79)	Mouse	C57BL/6	M	-	18–20	CS (Taconic Farms)	ND	5 to 12	America
Son et al. (80)	Mouse	C57BL/6 J	M and F	8	-	CS (OrientBio)	ND	12	Asia
Takanashi et al. (81)	Mouse	-	-	-	-	-	ND	5 to 17	Asia
Wang et al. (82)	Mouse	C57BL/6	M	8–10	23–26	BLF (Experimental Animal Center of Peking University)	40	10	Asia
Balkrishna et al. (83)	Mouse	CD-1	M	6–10	20–25	CS (Hylasco Biotechnology Pvt)	ND	5 to 6	Asia
Biggerstaff et al. (84)	Mouse	C57BL/6	M	8–12	28–34	BLF (Victoria University of Wellington breeding colonies)	ND	6 to 7	Oceania, Europe and America
Chen et al. (85)	Mouse	C57BL/6/BALB/c/NOD.CB17-Prkdcscid/NcrCr	F	7	-	BLF [Laboratory Animal Center (National Cheng Kung University)]	ND	5 to 10	Asia
Liang et al. (86)	Mouse	C57BL/6	M and F	8	15–20	-	ND	8	Asia
Lu et al. (87)	Mouse	C57BL/6	-	-	18–22	BLF (Nanjing QingLongShan)	ND	8 to 10	Asia
Inyang et al. (88)	Mouse	CD1	M and F	4	-	CS (Envigo)	ND	4 to 6	America
Kaur and Muthuraman (89)	Mouse	Swiss Albino	M	40	20–25	-	48	8	Asia
Mao et al. (90)	Mouse	CD1/DNMT3aKO	M	8–10	-	CS (Charles River Laboratory)	ND	8 to 12	America and Asia
Ramakrishna et al. (91)	Mouse	C57BL6/J/129S6/SvEvTac	M and F	-	-	CS (Jackson Laboratories and Taconic)	ND	8	America
Slivicki et al. (92)	Mouse	C57BL/6 J	M	12–14	-	CS (Jackson Laboratory)	ND	5 to 6	America
Tonello et al. (93)	Mouse	CD1	M and F	8–10	-	CS (Charles River Laboratory)	ND	3 to 6	America
Lin et al. (94)	Mouse	B6.129P2-CNR2 (tm1Dgen/J)/C57BL/6 J	M	-	25–33	BLF (Indiana University)/CS (Jackson Laboratory)	ND	6 to 8	America
**Characterization of the population: RATS/MICE**									
Kim et al. (95)	Rat/Mouse	Sprague–Dawley/Axin2-LacZ knock-in	M	-	200–350/20–30	CS (Harlan Sprague Dawley Company/Jackson Laboratory)	ND	6	America
Huynh et al. (96)	Rat/Mouse	Sprague–Dawley/C57BL/6	M	-	200–300/−	CS (Envigo)	ND	8	America
Chen et al. (97)	Rat/Mouse	Sprague–Dawley/C57BL/6	M	-	220–250/−	CS (Harlan Sprague Dawley Company)/−	122	16 to 29	America and Asia
Nie et al. (98)	Rat/Mouse	Sprague–Dawley/−	M	-	220–250	BLF [Institute of Experimental Animals of Sun Yat-sen University (R)]/CS [Jackson Laboratory (M)]	ND	6 to 12	Asia

BLF, bred at the local animal facility; CS, commercial supplier; F, female; M, male; ND, not mentioned.

In several studies we noticed the expression “adult” to define the age of the animals and, most important, in 70.5% of the studies using rats, and 37.9% of the studies using mice, did not even report the age. Most articles reported the weight of the animals, 93.2 %and 55.2% for rats and mice, respectively. However, only 22.7% of the publications using rats and 24.1% of the publications using mice reported the weight and age of the animals (Table 1).

Regarding the number of animals used in each study, 34.1% of the publications reported the total number of rats and 24.1% reported the total number of mice. Almost all publications (100% for rats and 96.6% for mice) reported the number of animals in each experimental group (Table 1).

Regarding to the continent where the studies were performed, the majority of studies were carried out in Asia (61.4% for rats and 51.7% for mice), followed by America (29.5% for rats and 51.7% for mice). A reduced number of articles performed the studies in more than one continent (6.8% for rats and 10.3% for mice; Table 1).

In terms of the supplier of rats, in 50% of the articles the animals were bought from commercial suppliers and 50% were bred at the local facilities. Regarding to the supplier of mice, 68% of articles used animals from commercial supplier and 32% of articles the animals were bred at the local facility. However, 14.3% of the publications did not specify the provider that was used to acquire the animals (Table 1).

### 3.3 Induction of CIPN

Nearly all studies (69 out of 70) selected the intraperitoneal (i.p.) route of paclitaxel administration over the intravenous (i.v.) route for the induction of the CIPN model. In rat studies, 36 out of 44 papers (81.8%), the most often employed paclitaxel doses were between 2 and 8 mg/kg. Only 8 publications (18.2%) used a dose lower than 2 mg/kg, and 1 article (2.3%) used a dosage more than 8 mg/kg. Noteworthy, 2 publications (4.5%) used more than one dose. Regarding to the mouse studies, mostly publications (96.6%) employed paclitaxel doses were between 2 and 8 mg/kg. Only 1 publication used a dose higher than 8 mg/kg (Table 2).

**Table 2:** Summary of the methods used for the induction of CIPN (paclitaxel dose, frequency of administration, vehicle, injection volume and duration of CIPN).

REF	Species	Dose (mg/kg)	Frequency of administration	Vehicle	Injection volume	Duration of CIPN (days)
**Induction of CIPN: RATS**						
Li et al. (32)	Rat	2	4 alternate days	10% DMSO, 20% PEG300 and 10% Tween 80 in saline	NM	21
Ma et al. (33)	Rat	2	4 alternate days	Cremophor EL and dehydrated ethanol diluted in normal saline	NM	14
Nasser et al. (34)	Rat	2	4 alternate days	Saline	NM	29
Sezer et al. (35)	Rat	4	4 alternate days	Ethanol and Cremophor EL (1:1) diluted in saline	NM	39
Wang et al. (36)	Rat	2	4 alternate days	DMSO	NM	14
Alkislar et al. (37)	Rat	2	4 alternate days	Cremophor EL, ethanol and saline (1:1:18)	NM	34
Chen et al. (38)	Rat	2	4 alternate days	10% DMSO, 40% PEG300 and 5% Tween 80 in saline	NM	21
Chou et al. (39)	Rat	2	4 alternate days	0.9% saline	NM	14
Garrido-Suárez et al. (40)	Rat	2	4 alternate days	Ethanol and Cremophor EL (50:50) diluted in saline	1 mL/kg	35
Ilari et al. (41)	Rat	2	7 alternate days	Saline	NM	15
Ma et al. (42)	Rat	2	4 alternate days	Cremophor EL and dehydrated ethanol (1:1) diluted in normal saline	NM	21
Meregalli et al. (43)	Rat	10	4 consecutive weeks	10% Tween 80, 10% absolute ethanol and 80% saline solution	NM	44
Semis et al. (44)	Rat	2	5 consecutive days	Saline	0.2 mL per animal	15
Wang et al. (45)	Rat	8	3 alternate days	Saline	NM	14
Zhang et al. (46)	Rat	2	4 alternate days	Anhydrous alcohol and hydrogenated castor oil (1:1) diluted in 0.9% saline	NM	35
Zhong et al. (47)	Rat	2	4 alternate days	Cremophor EL and absolute ethanol (1:1) diluted in saline	NM	28
Brewer et al. (48)	Rat	1	4 alternate days	0.9% saline	NM	45
Costa-Pereira et al. (21)	Rat	2	4 alternate days	4% DMSO	NM	28
Costa-Pereira et al. (22)	Rat	2	4 alternate days	4% DMSO	NM	28
Ferrari et al. (49)	Rat	1	4 alternate days	NM	NM	33
Hacimuftuoglu et al. (50)	Rat	2	4 alternate days	1 mL distilled water	NM	51
Huang et al. (51)	Rat	8	3 alternate days	Saline	NM	21
Kamata et al. (52)	Rat	2 and 4	4 alternate days	Cremophor EL and saline (1:2)	NM	14
Kim et al. (53)	Rat	2	4 alternate days	4% DMSO and 4% Tween 80 diluted in saline	NM	20
Liu et al. (54)	Rat	1	4 alternate days	Cremophor EL and saline (1:2)	NM	21
Wang et al. (55)	Rat	2	4 alternate days	Cremophor EL and ethanol (1:1) diluted in saline	NM	35
Zhang et al. (56)	Rat	2	4 alternate days	Saline	NM	35
Zhao et al. (57)	Rat	2	4 alternate days	PBS	NM	15
Zhou et al. (58)	Rat	2	4 alternate days	4% DMSO and 4% Tween 80 diluted in saline	NM	21
Zhou et al. (59)	Rat	2	4 alternate days	4% DMSO and 4% Tween 80 diluted in saline	NM	21
Li et al.	Rat	2	4 alternate days	0.9% saline	NM	14
Li et al. (60)	Rat	2	4 alternate days	Cremophor EL and ethanol (1:1)	NM	14
Sivanesan et al. (61)	Rat	1.5	4 alternate days	DMSO, 70% ethanol, and 0.9% saline	NM	30
Wu et al. (62)	Rat	1	4 consecutive days	NM	1 mL/kg	14
Wu et al. (63)	Rat	2	4 alternate days	DMSO diluted in saline	NM	21
Al-Mazidi et al. (64)	Rat	1	4 alternate days	Cremophor EL and ethanol (1:1)	NM	33
Ba et al. (65)	Rat	2	4 alternate days	DMSO diluted in saline	NM	20
Legakis et al. (66)	Rat	0.67, 2.0 and 6.0	4 alternate days	8.3% Ethanol, 8.3% Cremophor EL and 83.4% saline	2 mL/kg	29
Vitet et al. (67)	Rat	1	4 alternate days	10% Cremophor EL diluted in saline	NM	36
Zhang et al. (68)	Rat	2	4 alternate days	Cremophor EL and saline (1:2)	NM	47
**Induction of CIPN: MICE**						
Balkrishna et al. (69)	Mouse	2	6 consecutive days	Saline	NM	20
Cristiano et al. (70)	Mouse	8	4 alternate days	Saline	100 L per animal	14
Ezaka et al. (71)	Mouse	4	4 alternate days	Ethanol and Cremophor EL (1:1) diluted in normal saline (1:4)	NM	28
Karmakar et al. (72)	Mouse	2	5 consecutive days	NM	2 mL/kg	14
Lin et al. (73)	Mouse	4	4 alternate days	Cremophor EL, ethanol and 0.9% saline (1:1:18)	NM	26
Park et al. (74)	Mouse	4	5 consecutive days	5% DMSO	NM	44
Paton et al. (75)	Mouse	4	4 alternate days	Absolute ethanol, Cremophor EL and 0.9% saline (1:1:18)	NM	38
Caillaud et al. (76)	Mouse	8	4 alternate days	200 proof ethanol, kolliphor and distilled water (1:1:18)	NM	22
Caillaud et al. (77)	Mouse	8	4 alternate days	200 proof ethanol, kolliphor and distilled water (1:1:18)	NM	28
Cuozzo et al. (78)	Mouse	8	4 alternate days	Cremophor EL and absolute ethanol (1:1) diluted in 0.9% saline	NM	14
Foss et al. (79)	Mouse	8	4 alternate days	Saline	NM	28
Son et al. (80)	Mouse	2	4 times at 3-day intervals	NM	NM	20
Takanashi et al. (81)	Mouse	4	5 consecutive days	10% Cremophor EL and 10% ethanol diluted in saline	NM	28
Wang et al. (82)	Mouse	2	4 alternate days	Anhydrous alcohol and hydrogenated castor oil (1:1) diluted in 0.9% saline	NM	14
Balkrishna et al. (83)	Mouse	2	6 consecutive days	Saline	NM	20
Biggerstaff et al. (84)	Mouse	4	4 alternate days	Ethanol, kolliphor and 0.9% saline (1:1:18)	NM	40
Chen et al. (85)	Mouse	4.5	4 alternate days	NM	NM	35
Liang et al. (86)	Mouse	4	4 alternate days	Cremophor EL and ethanol (1:1) diluted in saline	NM	24
Lu et al. (87)	Mouse	20	4 alternate days	Cremophor EL and ethanol (1:1) diluted in 0.9% saline	NM	28
Inyang et al. (88)	Mouse	4	4 alternate days	Ethanol and kolliphor EL (1:1) diluted in 0.9% saline	NM	30
Kaur and Muthuraman (89)	Mouse	2	5 consecutive days	NM	NM	16
Mao et al. (90)	Mouse	4	4 consecutive days	Cremophor EL and ethanol (1:1)	NM	21
Ramakrishna et al. (91)	Mouse	4	4 alternate days	Cremophor EL and ethanol diluted in saline	NM	15
Slivicki et al. (92)	Mouse	4	4 alternate days	Cremophor EL, ethanol and saline (1:1:18)	6.67 mL/kg	44
Tonello et al. (93)	Mouse	2	4 alternate days	Cremophor EL and 95% dehydrated ethanol (1:1) diluted in saline	NM	28
Lin et al. (94)	Mouse	4	4 alternate days	Cremophor EL	NM	27
**Induction of CIPN: RATS/MICE**						
Kim et al. (95)	Rat/Mouse	2 (R)/4 (M)	4 alternate days	4% DMSO and 4% Tween 80 in saline (R)/0.8% DMSO and 0.8% Tween 80 in saline (M)	1 mL/kg (R)/10 mL/kg (M)	40
Huynh et al. (96)	Rat/Mouse	2	4 alternate days	1 part Cremophor EL and ethanol (1:1)and 2 parts 0.9% saline, and 2 mg/mL sodium citrate	1 mL/kg	51
Chen et al. (97)	Rat/Mouse	2	4 alternate days	Cremophor EL and ethanol (1:1)	NM	20
Nie et al. (98)	Rat/Mouse	8 (R)/2 (M)	3 alternate days (Rat) 5 consecutive days (M)	0.9% saline	NM	14

R, rat; M, mouse; DMSO, dimethyl sulfoxide; PBS, phosphate buffer saline; NM, Not mentioned.

Regarding to the frequency of the administration of paclitaxel, in studies using rats, 84.1% of the papers indicated 4 alternate days. Likewise, in studies using mice, the majority of publications (68.9%) indicated the same induction scheme (Table 2).

Regarding the composition of the vehicle in rat studies, 36.4% used cremophor or its derivates in conjunction with other components, e.g., ethanol and tween 80, 22.7% used saline, 27.3% reported dimethyl sulfoxide (DMSO), and 9.1% stated that other vehicles were used. Two publications (4.5%) did not indicate the vehicle used to dissolve paclitaxel (Table 2). As to the composition of the vehicle in mouse studies, 58.6% used cremophor or its derivates, 17.2% used saline, 6.9% used DMSO and 3.4% used other vehicles. Four publications (13.8%) did not indicate the vehicle (Table 2) herein there was no control group for the vehicle used to prepare the paclitaxel solution.

Most of the articles using rats (86.4%) and mice (86.2%) did not report the injection volume. In rat studies, of those that reported (13.6%), 4 publications indicated a volume that varies according to weight and 1 publication indicated a fixed injection volume per rat. In mouse studies, of those that reported (13.8%), 3 articles indicated a volume that varies according to weight and 1 publication indicated a fixed injection volume per mouse (Table 2).

Regarding the time of CIPN, we categorized the articles into three groups: less than 14 days; between 14 and 28 days; more or equal to 28 days. In publications using rats, 23 articles (52.3%) reported a time of CIPN between 14 and 28 days, whereas 21 articles (47.7%) showed equal or more than 28 days of CIPN. In publications using mice, 16 articles (55.2%) reported a duration of CIN between 14 and 28 days, and 13 publications (44.8%) showed equal or more than 28 days of CIPN. Although we did not include studies with less than 11 days, in our analysis we did not find studies with less than 14 days of CIPN (Table 2).

### 3.4 Methods of study

We analyzed the studies to extract the data regarding the methods of study and the tissues under analysis. The results are shown in Table 3. The techniques most commonly used (90%; 63 studies) were behavioral evaluations both for validation of the induction of CIPN and for testing of various substances (referred as “Pharmacology” in Table 3) or interventions (referred as “Non-pharmacological approaches”). All the behavioral analysis included nociception tests (described in detail Table 3). Immunohistochemistry was a technique frequently used (44.3%; 31 studies), in conjunction with Western-blotting. Multiple biochemical methods were used, such as ELISA and HPLC. Cell cultures and histopathology analysis were also performed (about 17% of the studies). Regarding the tissues collected the peripheral nerves/dorsal root ganglion were frequently collected (64.3%; 45 studies), along with the spinal cord (61.5%; 43 studies), although 6 of those studies analyzed simultaneously the DRG and spinal cord. Only a few studies evaluated supraspinal structures (22.9%; 16 studies). Due to the large preponderance of behavioral tests, we extracted the data regarding the types of tests and, in the case of nociception tests, we analyzed the sensory modality evaluated. The most used behavioral tests (97.1%) were mechanical stimuli with the von Frey test being the most common (84.3%; Supplementary Table 1). Forty-six publications (65.7%) reported thermal nociception tests, 28.5% only used hot stimuli, 17.1% used just cold stimuli and 15.7% employed both thermal modalities. Only 1 article (1.4%) used a spontaneous pain test (Supplementary Table 2). Eleven studies (15.7%) reported other behavioral tests which assess depressive- and anxiety-like behaviors, and locomotor activity (Supplementary Table 3). Fifty-nine publications (84.3%) that were analyzed conducted behavior tests repeatedly during several timepoints. Only 15.7% of the publications tested behavior in a single day. Noteworthy, 2 publications showed behavioral tests at several timepoints, but only reported the statistical analysis of a specific timepoint.

**Table 3:** Overview of the main methods and tissues used in the analyzed studies.

REF	Methods and tissues
Balkrishna et al. (69)	Biochemistry and Histopathology-sciatic nerve. Cell cultures. Pharmacology-nociception.
Cristiano et al. (70)	RT-PCR-brain and spinal cord. Pharmacology: nociception, anxiety and depression; Western Blotting: spinal cord.
Ezaka et al. (71)	Cell cultures-primary DRG and brain neurons; Immunohistochemistry-skin; Histopathology: sciatic nerves; qRT-PCR: DRG; Mass spectroscopy: plasma, liver, spinal cord and brain; Pharmacology: nociception.
Karmakar et al. (72)	Pharmacology: nociception, motor coordination and locomotion.
Li et al. (3, 32)	Immunohistochemistry and Western-blotting-spinal cord; Pharmacology: nociception.
Lin et al. (73)	Immunohistochemistry: spinal cord, brain and skin; Pharmacology: nociception; RT-PCR-spinal cord.
Ma et al. (33)	Immunohistochemistry, ELISA, qRT-PCR, Western-blotting: DRGs; Pharmacology: nociception.
Nasser et al. (34)	Biochemistry, ELISA, Histopathology, qRT-PCR and Western blotting -sciatic nerve; Pharmacology: nociception and locomotion.
Park et al. (74)	Pharmacology: nociception, locomotion, anxiety and depression; Immunofluorescence, Western-blotting and electrophysiology—spinal cord.
Paton et al. (75)	Pharmacology: nociception.
Sezer et al. (35)	ELISA -sciatic nerve and spinal cord; Non-pharmacological approaches (BM-MSCs transplantation): nociception.
Wang et al. (36)	ELISA: spinal cord; Pharmacology: nociception; Western-blotting and immunohistochemistry: DRG and spinal cord.
Alkislar et al. (37)	Functional imaging (brain resting-state blood oxygen level); Pharmacology: nociception.
Caillaud et al. (76)	Electrophysiology: nerve conduction; Immunohistochemistry: skin; Histopathology: morphology and mitochondrial ultrastructure -sciatic nerve; Multiplex assays and qRT-PCR: DRG and spinal cord; Pharmacology: nociception and locomotion.
Caillaud et al. (77)	Cell culture; Electrophysiology: nerve conduction; qRT-PCR: DRG and spinal cord; Pharmacology: nociception and “spontaneous pain”, locomotion, and strength.
Chen et al. (38)	Immunohistochemistry and Western-blotting: spinal cord; Mitochondrial biogenesis (counting of mitochondrial DNA copy numbers): spinal cord; Pharmacology: nociception.
Chou et al. (39)	Non-pharmacological approaches (hyperbaric oxygen therapy): nociception; Immunohistochemistry: spinal cord and DRG.
Cuozzo et al. (78)	Pharmacology: nociception; ELISA: plasma; Immunohistochemistry: paw skin; Western-blotting: spinal cord and colon.
Foss et al. (79)	In vitro radioligand binding studies; Pharmacology: nociception.
Garrido-Suárez et al. (40)	Histopathology: paw skin; Pharmacology: nociception.
Ilari et al. (41)	Biochemistry and Western-blotting: spinal cord; Pharmacology: nociception.
Kim et al. (95)	Biochemistry, Western blotting, qRT-PCR and Cell culture: DRG; Pharmacology: nociception and sedation.
Ma et al. (42)	Histopathology, Immunohistochemistry, Western-blotting and ELISA: DRG; Pharmacology: nociception.
Meregalli et al. (43)	Electrophysiology: sensory nerve conduction and sensory nerve action potential; Histopathology: sciatic and caudal nerves and DRG; Pharmacology: nociception
Semis et al. (44)	Biochemistry and RT-PCR: sciatic nerve; Pharmacology: nociception and locomotion.
Son et al. (80)	Cell culture; Immunoblotting; Pharmacology: nociception.
Takanashi et al. (81)	Pharmacology: nociception; Immunohistochemistry: spinal cord and brain.
Wang et al. (45)	Immunohistochemistry and Immunoprecipitation, qRT-PCR, Western blotting: spinal cord; Pharmacology: nociception.
Wang et al. (82)	ELISA, qRT-PCR and Western-blotting: DRG; Pharmacology: nociception.
Zhang et al. (46)	Pharmacology: nociception; Cell culture, qRT-PCR, Western-blotting and ELISA: DRG.
Zhong et al. (47)	Biochemistry: serum; Cell culture, RT-PCR, Cytokine array, Western-blotting and ELISA: DRG; Pharmacology: nociception.
Balkrishna et al. (83)	Biochemistry and Histopathology: sciatic nerve; ELISA: serum; Pharmacology: nociception.
Biggerstaff et al. (84)	Pharmacology: nociception, locomotion and anxiety.
Brewer et al. (48)	Non-pharmacological approaches (hyperbaric oxygen therapy): nociception and locomotion.
Chen et al. (85)	Human studies: Questionnaires and quantitative sensory testing (QST); ELISA: serum. Animal studies: Cell culture; qRT-PCR; ELISA: serum; Immunohistochemistry: DRG and hind paw skin; Histopathology: sciatic nerve; Pharmacology: nociception and motor coordination.
Costa-Pereira et al. (21)	Immunohistochemistry and Western-blotting: spinal cord; Pharmacology: nociception.
Costa-Pereira et al. (22)	Immunohistochemistry: spinal cord and brain; Pharmacology: nociception; HPLC: spinal cord.
Ferrari et al. (49)	Non-pharmacological approaches (several stressors): nociception.
Hacimuftuoglu et al. (50)	Histopathology: spinal cord and brain; Pharmacology: nociception.
Huang et al. (51)	Immunohistochemistry and Western-blotting: DRG; Pharmacology: nociception.
Huynh et al. (96)	Pharmacology: nociception.
Kamata et al. (52)	Western-blotting and Immunohistochemistry: spinal cord; Pharmacology: nociception.
Kim et al. (53)	Immunohistochemistry, qRT-PCR and Western-blotting: DRG; RNA sequencing analysis: DRG and spinal cord; Non-pharmacological approaches (circadian rhythm)—nociception.
Liang et al. (86)	qRT-PCR and RNA sequencing analysis: brain.
Liu et al. (54)	HPLC: spinal cord; Immunohistochemistry: spinal cord and brain; Pharmacology: nociception; Western-blotting: brain.
Lu et al. (87)	Cell culture: DRGs; Immunohistochemistry and Western-blotting: DRG and sciatic nerve; qRT-PCR: DRG; Pharmacology: nociception.
Wang et al. (55)	Biochemistry: spinal cord; Immunohistochemistry and Western-blotting: spinal cord; Pharmacology: nociception.
Zhang et al. (56)	Immunohistochemistry and Western-blotting: DRG; Non-pharmacological approaches (vagus nerve stimulation): nociception.
Zhao et al. (57)	ELISA: spinal cord and serum; Non-pharmacological approaches (electroacupuncture): nociception; Western-blotting: spinal cord.
Zhou et al. (58)	Immunohistochemistry and Western-blotting: spinal cord; Pharmacology: nociception.
Zhou et al. (59)	Immunohistochemistry and Western-blotting: spinal cord; Pharmacology: nociception.
Chen et al. (97)	Co-immunoprecipitation, qRT-PCR and Western-blotting: DRG and spinal cord. Electrophysiology: spinal cord slices; Pharmacology: nociception.
Inyang et al. (88)	Pharmacology: nociception.
Kaur and Muthuraman (89)	Biochemistry: muscle; Pharmacology: nociception.
Li et al.	Immunohistochemistry: DRG and spinal cord; Cell culture and Western-blotting: DRG; Pharmacology and non-Pharmacological approaches (electroacupuncture): nociception.
Li et al. (60)	ELISA, TUNEL, Western-blotting: DRG, spinal cord and brain; Histopathology and Immunohistochemistry: brain; Pharmacology: nociception
Mao et al. (90)	Cell culture, qRT-PCR, Western-blotting, Immunohistochemistry and Electrophysiology: DRG; DRG microinjection of siRNA or viral vectors; Pharmacology: nociception and “spontaneous pain”.
Ramakrishna et al. (91)	Isolation of mononuclear cells from spinal cord and brain; Flow cytometry analysis; Microbiome analyses; Non-Pharmacological approaches (gut microbiota): nociception.
Sivanesan et al. (61)	Non-Pharmacology (spinal cord stimulation): nociception, “spontaneous pain” and locomotion; RNA-seq libraries and qRT-PCR: spinal cord tissue.
Slivicki et al. (92)	Immunohistochemistry: brain; Non-Pharmacology (running well activity): nociception.
Tonello et al. (93)	Biochemistry: DRG; Immunohistochemistry: DRG and skin; Pharmacology: nociception; qRT-PCR: DRG.
Wu et al. (62)	Immunohistochemistry, Immunoblotting: spinal cord; Pharmacology: nociception; qRT-PCR: brain.
Wu et al. (63)	Biochemistry, qRT-PCR and Western blotting: DRG; Pharmacology: nociception and motor coordination.
Al-Mazidi et al. (64)	Biochemistry: plasma; Pharmacology: nociception.
Ba et al. (65)	ELISA and Western-blotting: DRG; Immunohistochemistry: spinal cord; Pharmacology: nociception and motor coordination.
Legakis et al. (66)	Non-Pharmacology (intracranial self-stimulation): nociception.
Lin et al. (94)	Cell culture and Biochemistry; Pharmacology: nociception.
Nie et al. (98)	Immunohistochemistry, RT-PCR, Electrophysiology and Western-blotting: DRG; Pharmacology: nociception.
Vitet et al. (67)	Pharmacology: nociception; Immunohistochemistry: sciatic nerve and hind paw skin.
Zhang et al. (68)	Non-pharmacological (electroacupuncture) and Pharmacology: nociception; Western-blotting: spinal cord.

The methods are listed in alphabetic order and the areas from which the tissues were collected are referred. BM-MSC, bone marrow-derived mesenchymal stem cells; DRG, Dorsal Root ganglion; ELISA, enzyme-linked immunosorbent assay; HPLC, High-performance liquid chromatography; RT-PCR, reverse transcription polymerase chain reaction; qRT-PCR, quantitative reverse transcription polymerase chain reaction; siRNA, small interfering RNA; TUNEL, terminal deoxynucleotidyl transferase dUTP nick end labeling.

### 3.5 Mechanisms and tissue sites under studies

We also analyzed the main mechanisms addressed in the retrieved publications, as well as the tissues and/or areas under study. The summary of the main mechanisms was shown in Table 4. Thirty of the analyzed studies (42.9%) focused on neuroinflammation (32–36, 39, 42–44, 46, 47, 51, 57, 60, 62, 64, 65, 70, 74, 76–78, 81–83, 85, 91, 93, 95, 98). The molecules under study were TNF-α, IL-1β, IL-6. Several of these studies analyzed the role of glial cells in driving neuroinflammation and the role of microglia (36, 39, 60, 62, 65, 91) and astrocytes (32, 36, 44, 57, 60, 65, 74, 81) was frequently evaluated. Oxidative stress (about 15.7%) was also analyzed (34, 41, 44, 58–59, 69, 71, 83, 89, 93). Several oxidative mediators, such as GSH, GSSG, SOD, CAT and MnSOD, were evaluated. Another set of studies focused on neurotransmitters and receptor systems with cannabinoids (8.6%) (37, 62, 70, 73, 79, 94), opioids (2.9%) (51, 75), monoamines (11.4%) (21, 22, 36, 38, 49, 54, 68, 87) and amino acids (5.7%) (46, 55, 84, 97) frequently studied. As to receptors, TRPV-1 studies (10%) stood out (36, 39, 52, 60, 63, 65, 80). It should be noted that several studies under analysis settled to analyze the mechanism underlying the effects of approaches such as “natural extracts” (72, 80, 96). Some studies were not aiming to establish the mechanisms of a drug but rather to apply in CIPN substances or approaches that are already been used in other pathologies, such as metformin (50), hyperbaric oxygen therapy (48), spinal cord stimulation (61), exercise (92), and intracranial self-stimulation (66). There were, however, studies which were not driven by a clear research hypothesis (67). Emerging mechanism in neuroscience research were evaluated namely in what concerns the gut-brain axis (78, 91) or circadian regulation (53).

**Table 4:** Mechanisms evaluated in each of the analyzed studies, summarizing the results (increases—↑; decreases—↓) in levels or responses in the referred tissues/areas.

Mechanisms	Alterations detected in paclitaxel-induced neuropathy
Neuroinflammation	↑ TNF-α, IL-1 β and IL6/hippocampus; ↑ TNF-α, IL-1 β, iNOS and COX-2/spinal cord; ↓ PPAR-α/spinal cord (70).
	↑ GFAP/spinal cord (32, 74).
	↑ macrophages, TNF-α and IL-1 β/DRG (33).
	↑ myeloperoxidase and IL-20/sciatic nerve; ↓ secretory leukocyte protease inhibitor (SLPI)/sciatic nerve (34).
	↑ TNF-α in the spinal cord (35, 36).
	↑ Iba-1 and GFAP/spinal cord (36).
	↑ IL-17A, TNF-α, IFN-γ and Keratinocyte Cytokine/spinal cord (76).
	↑ PPAR-α/DRG (77).
	↑ OX42 and TLR4/spinal cord (39).
	↑ TNF-α, IL-1 β, and IL-6/serum; ↑COX-2 and iNOS/spinal cord (78).
	↑ phospho-NF-κB, MCP-1, and IL-1 β/DRG (95).
	↑ IL-1 β and TNF-α and number of neurons surrounded by GFAP-cells/DRG (42).
	↑ CD68 macrophage infiltration/distal caudal nerves (43).
	↑ GFAP, NF-kB, IL-1 β, TNF-α, COX-2 and nNOS expression/sciatic nerve (44).
	↑ GFAP/primary sensory cortex (81).
	↑ IL-1 β/DRG; ↓ IL-10 DRG (82).
	↑ TNF-α and IL-6/DRG; ↓IL-10/DRG (46).
	↑ TLR4 and NF-κB p65, IL-1 β and MCP-1/DRG (47).
	↑ TNF-α/serum (83).
	↑ IL-20, TNF-α and macrophages infiltration/DRG; ↑TNF-α, IL-1b, and IL-20/serum (85).
	↑ IL-1β and TNF-α/DRG (51).
	↑ GFAP, TLR4 and NF-κB p65/spinal cord; ↑ IL-1 β and TNF-α/spinal cord and serum (57).
	↑ TLR4 and MyD88/DRG; ↓ GFAP and OX-42/spinal cord.
	↑ microglia proliferation induced by gut microbiota/brainstem and spinal cord (91).
	↓ IL-6 and TNF-α induced by MMP9 antibody/DRG (93).
	↑ Iba-1, IL-6, and phosphorylation of NF-kB subunit p65/spinal cord (62).
	↑ IL-1 α, IL-1 β, IL-6, TNF-α, MCP-1/CCL2 and INF-γ/plasma (64).
	↑ GFAP, Iba-1, TNF-α, IL-1 β and IL-6/spinal cord (65).
	↓ IL-10 and IL-4/DRG (98).
Oxidative stress	↑ GSSG MDA/sciatic nerve; ↓ GSH/sciatic nerve (69).
	↑superoxide/primary cortical neurons; upregulation of genes for antioxidant proteins/DRG (71).
	↑ MDA/sciatic nerve; ↓ GSH/sciatic nerve (34).
	↑ GSH, GLT-1 and ratio MnSOD nitrated/MnSOD/spinal cord (41).
	↑ MDA/sciatic nerve; ↓ GSH, SOD, CAT and GPx/sciatic nerve (44).
	↑ MDA and GSSG sciatic nerve (83).
	↑ Nrf2 and HO-1/spinal cord (58, 59).
	↑ TBARS sciatic nerve/↓ GSH/sciatic nerve (89).
	↑ MDA/striatum, spinal cord and DRG; ↓SOD/same areas (60).
	↓ dihydroethidium (DHE) intensity and SOD1 induced by MMP9 mAb/DRG (93).
Cannabinoids	↓ CB1 receptor/spinal cord (70).
	CB2 agonists suppressed cold and mechanical behavioral hypersensitivity in CB2 mice; ↑ CB2/epidermal Langerhans cells (73).
	Tetrahydrocannabinol (THC) reduces cold behavioral hypersensitivity; disconnection of hyperconnectivity patterns in brain areas (37).
	Cannabidiol and its structure analog KLS13019 prevent mechanical hypersensitivity (79).
	↑ colocalization of CB2 on reactive microglia/spinal dorsal horn; selective CB2 agonist MDA7, decreased neuroinflammation and prevent mechanical behavioral hypersensitivity (62).
	CB2 receptor agonist LY2828360 suppress mechanical and cold behavioral hypersensitivity (94).
Opioids	KOR agonists more potently reduce mechanical and thermal behavioral hypersensitivity compared to morphine (75).
	↓ mechanical hypersensitivity induced by JTC-801, a nociception/orphanin peptide (NOP) receptor antagonist (51).
Monoamines	Duloxetine-induced ↓ GFAP, Iba-1, CGRP and SP/spinal cord and or DRG; Duloxetine-induced dose-dependent decreases of mechanical and thermal behavioral hypersensitivity; (36).
	Duloxetine-induced neuroprotection/DRG and sciatic nerve; Duloxetine-induced decreases of mechanical and thermal hypersensitivity (87).
	↑ DBH/spinal cord; ↑ α_2_ adrenoreceptor-induced reduction of mechanical hypersensitivity (21).
	↑ serotonin, 5-HT3 receptor/spinal cord; ↑ numbers of serotoninergic neurons/RVM (22).
	↑ serotonin and 5-HT3 receptor function/spinal cord (54).
	↓ mechanical hyperalgesia induced by β2-adrenergic receptor antisense oligodeoxynucleotide (49).
	↓ in β2-adrenergic receptors/spinal cord (38).
	↓ mechanical hypersensitivity induced by 8-OH-DPAT (5-HT1A receptor agonist) (68).
Amino acids	↓ of GluA2/DRG (46).
	↑ VGLUT2 receptor in presynaptic neurons/spinal cord (55).
	↑ glutamatergic nociceptive input to spinal neurons with involvement of NMDA receptors in primary afferent terminals (97).
	↑ mechanical and thermal hypersensitivities induced by gabapentin (84).
TRPV1	↑ TRPV1/spinal cord (39, 52, 60).
	↑ TRPV1/spinal IB4 and CGRP neurons (36).
	↑ TRPV1/DRG (60, 63, 65).

CAT, catalase; CB1, cannabinoid receptor 1; CB2, cannabinoid receptor 2; CCL2, C-C motif chemokine ligand 2; CGRP, calcitonin gene-related peptide; COX2, cyclooxygenase-2; DBH, dopamine-β-hydroxylase; DRG, dorsal root ganglion; GFAP, glial fibrillary acidic protein; GLT-1, glutamate transporter; GluA2, alpha-amino-3-hydroxy-5-methyl-4-isoxazole-propionic acid receptor 2; GPx, glutathione peroxidase; GSH, reduced glutathione; GSSG, oxidized glutathione; IB4, isolectin B4; Iba-1, ionized calcium binding adaptor molecule 1; IFN-γ, interferon-gamma; IL1 β, interleukin-1 beta; IL4, interleukin-4; IL6, interleukin-6; IL-17A, interleukin-17A; IL20, interleukin-20; iNOS, inducible nitric oxide synthase; KOR, kappa opioid receptor; MCP-1, Monocyte chemoattractant protein-1; MDA, malondialdehyde; MnSOD, manganese superoxide dismutase; MyD88, Myeloid differentiation primary response 88; NF-κB, nuclear factor kappa-B; NMDA, N-metil D-aspartato; PPAR-α, peroxisome proliferator-activated receptor alpha; RVM, rostralventromedial medulla; SOD, superoxide dismutase; SP, substance P; TBARS, thiobarbituric acid reactive substance; TLR4, toll-like receptor 4; TNF-α, tumor necrosis factor alpha; TRPV1, transient receptor potential vanilloid 1; VGLUT2, vesicular glutamate transporter.

As to the tissue sites under study, the peripheral targets stood out, both in what concerns peripheral fibers in the sciatic nerve (34, 44, 69, 83, 89) and neurons at the dorsal root ganglia (33, 42, 46, 47, 51, 60, 63, 65, 71, 77, 82, 87, 93, 95, 98). At the central nervous system the focus was the spinal cord (21, 22, 32, 35, 36, 38, 39, 41, 52, 54, 55, 57–60, 62, 65, 70, 74, 76, 78, 91) and only a few studies approached areas of the descending pain control system, such as the rostroventromedial medulla (22, 37), the somatosensory cortex (81) and the prefrontal cortex (86) along with areas whose main function is not pain control such as the hippocampus (92).

### 3.6 Assessment of the reporting quality

#### 3.6.1 ARRIVE guidelines implementation

A global rating of strong was attributed to studies with a score higher than 15 (12.9%), moderate for a score between 10 and 15 (78.6%) and weak for those that scored under 10 (8.6%). Generally, the quality of the studies was moderate (Supplementary Table 4).

Five (7.1%) (32, 76, 78, 79, 97) of the 70 analyzed studies referred using the ARRIVE guidelines. The sub-items that the studies score high were the sub-item 1a (description of the experimental groups to be compared; 95.7%), 1b (definition of the experimental unit; 100%) and 7a (description of the statistical methods used; 95.7%). The most incomplete or missing sub-items were 6b (outcome measure that was used to determine the sample size) and item 10b (presentation of the effect size with confidence of interval), which failed 98.6% and 100%, respectively.

Regarding the ARRIVE’s recommended items, the quality of studies was also generally moderate, since 80% of the studies were scored as moderate and 20% was scored as weak (Supplementary Table 5).

#### 3.6.2 Risk of bias analysis

The risk of bias was evaluated using the SYRCLE Risk of Bias tool for animal studies and the results are shown in Supplementary Table 6. Only 1 study out of the 70 analyzed, obtained 5 responses with low risk of bias and 5 with high risk or unclear. The remaining included studies presented most of the responses classified as high risk or unclear risk of bias. In detail, almost all studies showed a low risk of bias in components like groups similar at the baseline (80%) and selective reporting bias (93%). In addition, 59% presented low risk of bias in blinded outcome assessment domain. Most of the studies were scored as high risk or unclear risk of bias in several components, namely random group allocation, blinded group allocation, random housing, blinded intervention, random outcome assessment, reporting incomplete data and other sources of bias (Supplementary Table 6).

## 4 Discussion

The present study provides a large and comprehensive summary of the recent research in preclinical CIPN models. We elected CIPN because it is a major problem both for cancer patients and cancer survivors and also due to our research interests in animal models of CIPN (21–23).

Research would benefit if animal models used in preclinical research are clinically relevant. In the case of paclitaxel-induced peripheral neuropathy models, at least one of the cancers treated with paclitaxel (breast cancer) is much more prevalent in females (99) cancer prevalence increases with age (100, 101). However, the characterization of sex and age of the animal population of the 70 analyzed studies shows that most studies were performed in young adult males. Only 4 studies aimed to investigate sex differences during CIPN (49, 66, 86, 88) which indicates that some researchers are concerned with the translational perspectives of the CIPN research. As to age, and besides the lack of studies in old animals, some studies do not even report the age of the animals used or only refer their weight, with need to indirectly infer the age of the animals. The importance of the use of aged animals should be highlighted due to the abovementioned age-related increase in cancer prevalence (100, 101) and since other comorbidities, such as depression, with an impact at CIPN often appear in aging populations (102). Collectively, and regarding sex and age issues, we consider that there are still challenges in translation of the results of the CIPN studies. Similar concerns about sex and age in pain types other than CIPN were previously pinpointed (24, 25). Those challenges in translation may be increased by additional issues of the animal population used in the studies namely lack of heterogeneity of the genetic background along with lack of report of the origin (animal supplier) and genetic background.

As to the features of CIPN induction, most studies aim to reproduce a chemotherapy cycle, and most paclitaxel doses were between 2–8 mg/Kg. A concern emerging from analyzing the studies relates to CIPN induction due to the heterogeneity of paclitaxel solvents, which was not always accompanied by the appropriate controls for the solvent. In several studies, the use of a control group for the solvent used in paclitaxel preparation was not even performed/reported. This is important because the solvents, such as DMSO at high concentrations, commonly used in CIPN studies, have significant neurotoxic effects (103). These neurotoxic effects can introduce important confounding factors. One of the settings of the present systematic review was the analysis of studies with CIPN induction ≥11 days and exclusion of studies with shorter timepoints. This time point was elected since the studies that validated the CIPN animal model evaluate putative clinically relevant pain-like behaviors at those time points the use of long post-induction periods may increase the translational perspectives of the studies (11, 104). Noteworthy, most of the studies under analysis in this systematic review used CIPN times between 14–28 days, which is a period in which mechanisms and neuroplastic changes underlying paclitaxel-induced neuropathy were established (21, 22, 105). However, with the improvement of early diagnosis of cancer and introduction of more effective treatments, besides the problem of CIPN during cancer treatment, another problem is CIPN-associated complaints by cancer survivors (3). These problems need better addressing since the longer time of CIPN study in the studies analyzed was 51 days (43, 50).

Regarding the methods used in the analyzed studies it should be highlighted the diversity of the techniques applied, namely behavioral analysis, histopathology, and biochemistry, which may be considered techniques with translational perspectives. Furthermore, almost all studies used diverse methods directed to enlighten a biological question. Curiously, a single study used brain imaging, which is a technique with a putative translational value (106). As to the behavioral analysis of nociception, most studies tested mechanical hypersensitivity. It should, however, be noted that the main complaints of the patients with CIPN are spontaneous pain and hypersensitivity related the thermal stimuli, including cold allodynia (1, 5, 6). Still regarding the behavioral studies, it should be noted that some studies aim to approach not only the nociceptive responses but also other responses that are interrelated with pain, such as spontaneous pain and emotions, which is important since pain is affected and has an impact in other functions which may be relevant in the translational perspective. Therefore, we conclude that the putative clinical relevance of the animal studies could be increased if the methods to study spontaneous pain will become more frequently used in the future.

As to the mechanisms underlying paclitaxel-induced peripheral neuropathy in the analyzed studies, it is interesting that besides the studies related to drugs frequently used in CIPN treatment, namely antidepressants with their action in serotonin and noradrenaline reuptake, the interest in neuroinflammation is increasing. It could be considered that this is in line with the research trends in other types of neuropathic pain, where inflammation and the role of the balance between pro- and anti-inflammatory cytokines is increasingly studied (107). Regarding the areas of the nervous system under analysis, almost all studies were focused on the peripheral fibers and, in a less extension, at the spinal cord. The supraspinal mechanisms of pain modulation during CIPN remain understudied in spite of the fact that pain is frequently associated with comorbidities, such as anxiety and depression, and in CIPN there are major neuroplastic changes in brain structures.

Regarding reporting and risk of bias, besides the abovementioned constraints in report (e.g., animal age, control groups and types of solvents), only 5 reports state that they were performed in accordance with the ARRIVE guidelines (Animal Research: Reporting of In Vivo Experiments). As to this and related to animal research, the importance of applying adequate experimental design and proper and detailed report, namely following the ARRIVE guidelines (28) needs to be considered to maximize the reproducibility of research, which is also important for the translational issues.

This systematic review includes 70 original papers and presents some limitations. Our first aim was to reflect about animal research on paclitaxel-induced neuropathy but due to the constraints of analyzing in detail large study samples, we excluded studies in which paclitaxel was combined with other therapeutical approaches. This analysis will be performed in a near future since the combination of cytostatic drugs with other approaches such as antibody therapy may decrease the cytostatic drug does or shorten the CIPN protocol. Also, we elected only paclitaxel due to its neurotoxic impact at the peripheral and central nervous system (108). We cannot, herein extrapolate the results of the present systematic review to other CIPN types. Furthermore, due to the exclusion/inclusion criteria and search in 2 databases the number of studies of paclitaxel-induced peripheral neuropathy may be underestimated. Finally, we did not analyze in detail the experimental design of the studies in what concerns the day of each behavioral test or the sequence of experiments because this was also underreported in several of the analyzed studies.

In conclusion, the present systematic review shows that there is a substantial effort in preclinical CIPN research. However, this systematic review also alerts to some potential problems related to underreporting which may mirror the poor experimental design. This could be overcome by a strict report of the ARRIVE guidelines, which is not requested by several journals along with implementation of the guidelines from PREPARE (Planning Research and Experimental Procedures on Animals: Recommendations for Excellence). The latter can substantially improve experimental planning since it takes into account aspects related to the formulation of study (e.g., literature searches, humane endpoints, and experimental design), dialog between scientists and colleagues from the animal facility (e.g., division of labor, education and training, and facility evaluation), and quality control of the components in the study (e.g., housing and husbandry, and necropsy) (109). The need to design studies which are representative of the problems of the CIPN patients, such as sex, age and pain types, needs to be considered. The use of correct terminology in the animal studies, avoiding the terms such as “pain” and “hyperalgesia”, and replacing by the hypersensitivity to noxious events, which has already discussed in other contexts (110), also needs to be considered by the authors and editorial managers of the journals. In conclusion, the detailed analysis of the animal studies of paclitaxel-induced peripheral neuropathy may alert to the importance of ameliorating the experimental design and report of the studies which is important for replication and translation of the results of animal studies into the clinical setting. This is relevant inasmuch that the aim of most of the analyzed studies was the clinical application of the results and since CIPN is a major clinical problem for cancer patients and cancer survivors.

## Data availability statement

The original contributions presented in the study are included in the article/Supplementary material, further inquiries can be directed to the corresponding author.

## Author contributions

CB: Data curation, Formal analysis, Investigation, Writing – review & editing. JC-P: Conceptualization, Data curation, Formal analysis, Investigation, Writing – original draft, Writing – review & editing. IT: Conceptualization, Data curation, Funding acquisition, Investigation, Supervision, Writing – original draft, Writing – review & editing.
