# Supplementary material for: Preclinical research in paclitaxel-induced neuropathic pain: a systematic review
Source: Front Vet Sci. 2023 Dec 18;10:1264668. doi: 10.3389/fvets.2023.1264668 (PMC10766764; doi:10.3389/fvets.2023.1264668)
Supplement: Supplementary file 4 [file Table_4.docx]

| **ARRIVE Essential 10** | | | | | | | | | | | | | | | | | | | | | | | | |
| --- | --- | --- | --- | --- | --- | --- | --- | --- | --- | --- | --- | --- | --- | --- | --- | --- | --- | --- | --- | --- | --- | --- | --- | --- |
| **REF** | **1a** | **1b** | **2a** | **2b** | **3a** | **3b** | **3c** | **4a** | **4b** | **5** | **6a** | **6b** | **7a** | **7b** | **8a** | **8b** | **9a** | **9b** | **9c** | **9d** | **10a** | **10b** | **Global Score** | ** |
| (Balkrishna et al., 2022) |  |  |  |  |  |  |  |  |  |  |  |  |  |  |  |  |  |  |  |  |  |  | **M** | **No** |
| (Cristiano et al., 2022) |  |  |  |  |  |  |  |  |  |  |  |  |  |  |  |  |  |  |  |  |  |  | **M** | **No** |
| (Ezaka et al., 2022) |  |  |  |  |  |  |  |  |  |  |  |  |  |  |  |  |  |  |  |  |  |  | **H** | **No** |
| (Karmakar et al., 2022) |  |  |  |  |  |  |  |  |  |  |  |  |  |  |  |  |  |  |  |  |  |  | **M** | **No** |
| (Li et al., 2022a) |  |  |  |  |  |  |  |  |  |  |  |  |  |  |  |  |  |  |  |  |  |  | **H** | **YES** |
| (Lin et al., 2022) |  |  |  |  |  |  |  |  |  |  |  |  |  |  |  |  |  |  |  |  |  |  | **M** | **No** |
| (Ma et al., 2022) |  |  |  |  |  |  |  |  |  |  |  |  |  |  |  |  |  |  |  |  |  |  | **M** | **No** |
| (Nasser et al., 2022) |  |  |  |  |  |  |  |  |  |  |  |  |  |  |  |  |  |  |  |  |  |  | **M** | **No** |
| (Park et al., 2022) |  |  |  |  |  |  |  |  |  |  |  |  |  |  |  |  |  |  |  |  |  |  | **L** | **No** |
| (Paton et al., 2022) |  |  |  |  |  |  |  |  |  |  |  |  |  |  |  |  |  |  |  |  |  |  | **M** | **No** |
| (Sezer et al., 2022) |  |  |  |  |  |  |  |  |  |  |  |  |  |  |  |  |  |  |  |  |  |  | **M** | **No** |
| (Wang et al., 2022) |  |  |  |  |  |  |  |  |  |  |  |  |  |  |  |  |  |  |  |  |  |  | **L** | **No** |
| (Alkislar et al., 2021) |  |  |  |  |  |  |  |  |  |  |  |  |  |  |  |  |  |  |  |  |  |  | **M** | **No** |
| (Caillaud et al., 2021a) |  |  |  |  |  |  |  |  |  |  |  |  |  |  |  |  |  |  |  |  |  |  | **H** | **YES** |
| (Caillaud et al., 2021b) |  |  |  |  |  |  |  |  |  |  |  |  |  |  |  |  |  |  |  |  |  |  | **M** | **No** |
| (Chen et al., 2021) |  |  |  |  |  |  |  |  |  |  |  |  |  |  |  |  |  |  |  |  |  |  | **M** | **No** |
| (Chou et al., 2021) |  |  |  |  |  |  |  |  |  |  |  |  |  |  |  |  |  |  |  |  |  |  | **M** | **No** |
| (Cuozzo et al., 2021) |  |  |  |  |  |  |  |  |  |  |  |  |  |  |  |  |  |  |  |  |  |  | **M** | **YES** |
| (Foss et al., 2021) |  |  |  |  |  |  |  |  |  |  |  |  |  |  |  |  |  |  |  |  |  |  | **H** | **YES** |
| (Garrido-Suárez et al., 2021) |  |  |  |  |  |  |  |  |  |  |  |  |  |  |  |  |  |  |  |  |  |  | **M** | **No** |
| (Ilari et al., 2021) |  |  |  |  |  |  |  |  |  |  |  |  |  |  |  |  |  |  |  |  |  |  | **M** | **No** |
| (Kim et al., 2021) |  |  |  |  |  |  |  |  |  |  |  |  |  |  |  |  |  |  |  |  |  |  | **M** | **No** |
| (Ma et al., 2021) |  |  |  |  |  |  |  |  |  |  |  |  |  |  |  |  |  |  |  |  |  |  | **M** | **No** |
| (Meregalli et al., 2021) |  |  |  |  |  |  |  |  |  |  |  |  |  |  |  |  |  |  |  |  |  |  | **M** | **No** |
| (Semis et al., 2021) |  |  |  |  |  |  |  |  |  |  |  |  |  |  |  |  |  |  |  |  |  |  | **L** | **No** |
| (Son et al., 2021) |  |  |  |  |  |  |  |  |  |  |  |  |  |  |  |  |  |  |  |  |  |  | **M** | **No** |
| (Takanashi et al., 2021) |  |  |  |  |  |  |  |  |  |  |  |  |  |  |  |  |  |  |  |  |  |  | **L** | **No** |
| (Wang et al., 2021a) |  |  |  |  |  |  |  |  |  |  |  |  |  |  |  |  |  |  |  |  |  |  | **M** | **No** |
| (Wang et al., 2021b) |  |  |  |  |  |  |  |  |  |  |  |  |  |  |  |  |  |  |  |  |  |  | **M** | **No** |
| (Zhang et al., 2021) |  |  |  |  |  |  |  |  |  |  |  |  |  |  |  |  |  |  |  |  |  |  | **M** | **No** |
| (Zhong et al., 2021) |  |  |  |  |  |  |  |  |  |  |  |  |  |  |  |  |  |  |  |  |  |  | **M** | **No** |
| (Balkrishna et al., 2020) |  |  |  |  |  |  |  |  |  |  |  |  |  |  |  |  |  |  |  |  |  |  | **H** | **No** |
| (Biggerstaff et al., 2020) |  |  |  |  |  |  |  |  |  |  |  |  |  |  |  |  |  |  |  |  |  |  | **M** | **No** |
| (Brewer et al., 2020) |  |  |  |  |  |  |  |  |  |  |  |  |  |  |  |  |  |  |  |  |  |  | **M** | **No** |
| (Chen et al., 2020) |  |  |  |  |  |  |  |  |  |  |  |  |  |  |  |  |  |  |  |  |  |  | **M** | **No** |
| (Costa-Pereira et al., 2020a) |  |  |  |  |  |  |  |  |  |  |  |  |  |  |  |  |  |  |  |  |  |  | **M** | **No** |
| (Costa-Pereira et al., 2020b) |  |  |  |  |  |  |  |  |  |  |  |  |  |  |  |  |  |  |  |  |  |  | **M** | **No** |
| (Ferrari et al., 2020) |  |  |  |  |  |  |  |  |  |  |  |  |  |  |  |  |  |  |  |  |  |  | **M** | **No** |
| (Hacimuftuoglu et al., 2020) |  |  |  |  |  |  |  |  |  |  |  |  |  |  |  |  |  |  |  |  |  |  | **L** | **No** |
| (Huang et al., 2020) |  |  |  |  |  |  |  |  |  |  |  |  |  |  |  |  |  |  |  |  |  |  | **M** | **No** |
| (Huynh et al., 2020) |  |  |  |  |  |  |  |  |  |  |  |  |  |  |  |  |  |  |  |  |  |  | **M** | **No** |
| (Kamata et al., 2020) |  |  |  |  |  |  |  |  |  |  |  |  |  |  |  |  |  |  |  |  |  |  | **M** | **No** |
| (Kim et al., 2020) |  |  |  |  |  |  |  |  |  |  |  |  |  |  |  |  |  |  |  |  |  |  | **M** | **No** |
| (Liang et al., 2020) |  |  |  |  |  |  |  |  |  |  |  |  |  |  |  |  |  |  |  |  |  |  | **M** | **No** |
| (Liu et al., 2020) |  |  |  |  |  |  |  |  |  |  |  |  |  |  |  |  |  |  |  |  |  |  | **M** | **No** |
| (Lu et al., 2020) |  |  |  |  |  |  |  |  |  |  |  |  |  |  |  |  |  |  |  |  |  |  | **M** | **No** |
| (Wang et al., 2020) |  |  |  |  |  |  |  |  |  |  |  |  |  |  |  |  |  |  |  |  |  |  | **M** | **No** |
| (Zhang et al., 2020) |  |  |  |  |  |  |  |  |  |  |  |  |  |  |  |  |  |  |  |  |  |  | **M** | **No** |
| (Zhao et al., 2020) |  |  |  |  |  |  |  |  |  |  |  |  |  |  |  |  |  |  |  |  |  |  | **M** | **no** |
| (Zhou et al., 2020a) |  |  |  |  |  |  |  |  |  |  |  |  |  |  |  |  |  |  |  |  |  |  | **M** | **No** |
| (Zhou et al., 2020b) |  |  |  |  |  |  |  |  |  |  |  |  |  |  |  |  |  |  |  |  |  |  | **M** | **No** |
| (Chen et al., 2019) |  |  |  |  |  |  |  |  |  |  |  |  |  |  |  |  |  |  |  |  |  |  | **H** | **YES** |
| (Inyang et al., 2019) |  |  |  |  |  |  |  |  |  |  |  |  |  |  |  |  |  |  |  |  |  |  | **M** | **No** |
| (Kaur and Muthuraman, 2019) |  |  |  |  |  |  |  |  |  |  |  |  |  |  |  |  |  |  |  |  |  |  | **L** | **No** |
| (Li et al., 2019a) |  |  |  |  |  |  |  |  |  |  |  |  |  |  |  |  |  |  |  |  |  |  | **M** | **No** |
| (Li et al., 2019b) |  |  |  |  |  |  |  |  |  |  |  |  |  |  |  |  |  |  |  |  |  |  | **M** | **No** |
| (Mao et al., 2019) |  |  |  |  |  |  |  |  |  |  |  |  |  |  |  |  |  |  |  |  |  |  | **M** | **No** |
| (Ramakrishna et al., 2019) |  |  |  |  |  |  |  |  |  |  |  |  |  |  |  |  |  |  |  |  |  |  | **M** | **No** |
| (Sivanesan et al., 2019) |  |  |  |  |  |  |  |  |  |  |  |  |  |  |  |  |  |  |  |  |  |  | **H** | **No** |
| (Slivicki et al., 2019) |  |  |  |  |  |  |  |  |  |  |  |  |  |  |  |  |  |  |  |  |  |  | **M** | **No** |
| (Tonello et al., 2019) |  |  |  |  |  |  |  |  |  |  |  |  |  |  |  |  |  |  |  |  |  |  | **M** | **No** |
| (Wu et al., 2019a) |  |  |  |  |  |  |  |  |  |  |  |  |  |  |  |  |  |  |  |  |  |  | **H** | **No** |
| (Wu et al., 2019b) |  |  |  |  |  |  |  |  |  |  |  |  |  |  |  |  |  |  |  |  |  |  | **M** | **No** |
| (Al-Mazidi et al., 2018) |  |  |  |  |  |  |  |  |  |  |  |  |  |  |  |  |  |  |  |  |  |  | **H** | **No** |
| (Ba et al., 2018) |  |  |  |  |  |  |  |  |  |  |  |  |  |  |  |  |  |  |  |  |  |  | **M** | **No** |
| (Legakis et al., 2018) |  |  |  |  |  |  |  |  |  |  |  |  |  |  |  |  |  |  |  |  |  |  | **M** | **No** |
| (Lin et al., 2018) |  |  |  |  |  |  |  |  |  |  |  |  |  |  |  |  |  |  |  |  |  |  | **M** | **No** |
| (Nie et al., 2018) |  |  |  |  |  |  |  |  |  |  |  |  |  |  |  |  |  |  |  |  |  |  | **M** | **No** |
| (Vitet et al., 2018) |  |  |  |  |  |  |  |  |  |  |  |  |  |  |  |  |  |  |  |  |  |  | **M** | **No** |
| (Zhang et al., 2018) |  |  |  |  |  |  |  |  |  |  |  |  |  |  |  |  |  |  |  |  |  |  | **M** | **No** |

Abbreviations: M – Moderate; H – High; L – Low.

1a and 1b – Study design; 2a and 2b – Sample size; 3a – 3c – Inclusion and exclusion criteria; 4a and 4b – Randomisation; 5 – Blinding; 6a and 6b – Outcome measures; 7a and 7b – Statistical methods; 8a and 8b - Experimental animals; 9a – 9d – Experimental procedures; 10a and 10b – Results. ** “Does the article mention/follow the Arrive guidelines?”

**Supplementary Table 4 -** Summary of the ARRIVE guidelines implementation.
