# Supplementary material for: Preclinical research in paclitaxel-induced neuropathic pain: a systematic review
Source: Front Vet Sci. 2023 Dec 18;10:1264668. doi: 10.3389/fvets.2023.1264668 (PMC10766764; doi:10.3389/fvets.2023.1264668)
Supplement: Supplementary file 5 [file Table_5.docx]

| **The Recommended Set** | | | | | | | | | | | | | | | | | |
| --- | --- | --- | --- | --- | --- | --- | --- | --- | --- | --- | --- | --- | --- | --- | --- | --- | --- |
| **REF** | **11** | **12a** | **12b** | **13** | **14** | **15** | **16a** | **16b** | **16c** | **17a** | **17b** | **18** | **19** | **20** | **21a** | **21b** | **Global Score** |
| (Balkrishna et al., 2022) |  |  |  |  |  |  |  |  |  |  |  |  |  |  |  |  | **M** |
| (Cristiano et al., 2022) |  |  |  |  |  |  |  |  |  |  |  |  |  |  |  |  | **M** |
| (Ezaka et al., 2022) |  |  |  |  |  |  |  |  |  |  |  |  |  |  |  |  | **M** |
| (Karmakar et al., 2022) |  |  |  |  |  |  |  |  |  |  |  |  |  |  |  |  | **M** |
| (Li et al., 2022a) |  |  |  |  |  |  |  |  |  |  |  |  |  |  |  |  | **M** |
| (Lin et al., 2022) |  |  |  |  |  |  |  |  |  |  |  |  |  |  |  |  | **M** |
| (Ma et al., 2022) |  |  |  |  |  |  |  |  |  |  |  |  |  |  |  |  | **M** |
| (Nasser et al., 2022) |  |  |  |  |  |  |  |  |  |  |  |  |  |  |  |  | **L** |
| (Park et al., 2022) |  |  |  |  |  |  |  |  |  |  |  |  |  |  |  |  | **M** |
| (Paton et al., 2022) |  |  |  |  |  |  |  |  |  |  |  |  |  |  |  |  | **M** |
| (Sezer et al., 2022) |  |  |  |  |  |  |  |  |  |  |  |  |  |  |  |  | **M** |
| (Wang et al., 2022) |  |  |  |  |  |  |  |  |  |  |  |  |  |  |  |  | **M** |
| (Alkislar et al., 2021) |  |  |  |  |  |  |  |  |  |  |  |  |  |  |  |  | **M** |
| (Caillaud et al., 2021a) |  |  |  |  |  |  |  |  |  |  |  |  |  |  |  |  | **M** |
| (Caillaud et al., 2021b) |  |  |  |  |  |  |  |  |  |  |  |  |  |  |  |  | **M** |
| (Chen et al., 2021) |  |  |  |  |  |  |  |  |  |  |  |  |  |  |  |  | **L** |
| (Chou et al., 2021) |  |  |  |  |  |  |  |  |  |  |  |  |  |  |  |  | **M** |
| (Cuozzo et al., 2021) |  |  |  |  |  |  |  |  |  |  |  |  |  |  |  |  | **M** |
| (Foss et al., 2021) |  |  |  |  |  |  |  |  |  |  |  |  |  |  |  |  | **M** |
| (Garrido-Suárez et al., 2021) |  |  |  |  |  |  |  |  |  |  |  |  |  |  |  |  | **M** |
| (Ilari et al., 2021) |  |  |  |  |  |  |  |  |  |  |  |  |  |  |  |  | **M** |
| (Kim et al., 2021) |  |  |  |  |  |  |  |  |  |  |  |  |  |  |  |  | **M** |
| (Ma et al., 2021) |  |  |  |  |  |  |  |  |  |  |  |  |  |  |  |  | **M** |
| (Meregalli et al., 2021) |  |  |  |  |  |  |  |  |  |  |  |  |  |  |  |  | **M** |
| (Semis et al., 2021) |  |  |  |  |  |  |  |  |  |  |  |  |  |  |  |  | **L** |
| (Son et al., 2021) |  |  |  |  |  |  |  |  |  |  |  |  |  |  |  |  | **M** |
| (Takanashi et al., 2021) |  |  |  |  |  |  |  |  |  |  |  |  |  |  |  |  | **M** |
| (Wang et al., 2021a) |  |  |  |  |  |  |  |  |  |  |  |  |  |  |  |  | **L** |
| (Wang et al., 2021b) |  |  |  |  |  |  |  |  |  |  |  |  |  |  |  |  | **L** |
| (Zhang et al., 2021) |  |  |  |  |  |  |  |  |  |  |  |  |  |  |  |  | **L** |
| (Zhong et al., 2021) |  |  |  |  |  |  |  |  |  |  |  |  |  |  |  |  | **M** |
| (Balkrishna et al., 2020) |  |  |  |  |  |  |  |  |  |  |  |  |  |  |  |  | **M** |
| (Biggerstaff et al., 2020) |  |  |  |  |  |  |  |  |  |  |  |  |  |  |  |  | **M** |
| (Brewer et al., 2020) |  |  |  |  |  |  |  |  |  |  |  |  |  |  |  |  | **M** |
| (Chen et al., 2020) |  |  |  |  |  |  |  |  |  |  |  |  |  |  |  |  | **M** |
| (Costa-Pereira et al., 2020a) |  |  |  |  |  |  |  |  |  |  |  |  |  |  |  |  | **M** |
| (Costa-Pereira et al., 2020b) |  |  |  |  |  |  |  |  |  |  |  |  |  |  |  |  | **M** |
| (Ferrari et al., 2020) |  |  |  |  |  |  |  |  |  |  |  |  |  |  |  |  | **M** |
| (Hacimuftuoglu et al., 2020) |  |  |  |  |  |  |  |  |  |  |  |  |  |  |  |  | **M** |
| (Huang et al., 2020) |  |  |  |  |  |  |  |  |  |  |  |  |  |  |  |  | **M** |
| (Huynh et al., 2020) |  |  |  |  |  |  |  |  |  |  |  |  |  |  |  |  | **M** |
| (Kamata et al., 2020) |  |  |  |  |  |  |  |  |  |  |  |  |  |  |  |  | **M** |
| (Kim et al., 2020) |  |  |  |  |  |  |  |  |  |  |  |  |  |  |  |  | **M** |
| (Liang et al., 2020) |  |  |  |  |  |  |  |  |  |  |  |  |  |  |  |  | **M** |
| (Liu et al., 2020) |  |  |  |  |  |  |  |  |  |  |  |  |  |  |  |  | **M** |
| (Lu et al., 2020) |  |  |  |  |  |  |  |  |  |  |  |  |  |  |  |  | **L** |
| (Wang et al., 2020) |  |  |  |  |  |  |  |  |  |  |  |  |  |  |  |  | **M** |
| (Zhang et al., 2020) |  |  |  |  |  |  |  |  |  |  |  |  |  |  |  |  | **M** |
| (Zhao et al., 2020) |  |  |  |  |  |  |  |  |  |  |  |  |  |  |  |  | **L** |
| (Zhou et al., 2020a) |  |  |  |  |  |  |  |  |  |  |  |  |  |  |  |  | **M** |
| (Zhou et al., 2020b) |  |  |  |  |  |  |  |  |  |  |  |  |  |  |  |  | **M** |
| (Chen et al., 2019) |  |  |  |  |  |  |  |  |  |  |  |  |  |  |  |  | **M** |
| (Inyang et al., 2019) |  |  |  |  |  |  |  |  |  |  |  |  |  |  |  |  | **M** |
| (Kaur and Muthuraman, 2019) |  |  |  |  |  |  |  |  |  |  |  |  |  |  |  |  | **L** |
| (Li et al., 2019a) |  |  |  |  |  |  |  |  |  |  |  |  |  |  |  |  | **M** |
| (Li et al., 2019b) |  |  |  |  |  |  |  |  |  |  |  |  |  |  |  |  | **M** |
| (Mao et al., 2019) |  |  |  |  |  |  |  |  |  |  |  |  |  |  |  |  | **L** |
| (Ramakrishna et al., 2019) |  |  |  |  |  |  |  |  |  |  |  |  |  |  |  |  | **M** |
| (Sivanesan et al., 2019) |  |  |  |  |  |  |  |  |  |  |  |  |  |  |  |  | **M** |
| (Slivicki et al., 2019) |  |  |  |  |  |  |  |  |  |  |  |  |  |  |  |  | **M** |
| (Tonello et al., 2019) |  |  |  |  |  |  |  |  |  |  |  |  |  |  |  |  | **L** |
| (Wu et al., 2019a) |  |  |  |  |  |  |  |  |  |  |  |  |  |  |  |  | **L** |
| (Wu et al., 2019b) |  |  |  |  |  |  |  |  |  |  |  |  |  |  |  |  | **M** |
| (Al-Mazidi et al., 2018) |  |  |  |  |  |  |  |  |  |  |  |  |  |  |  |  | **M** |
| (Ba et al., 2018) |  |  |  |  |  |  |  |  |  |  |  |  |  |  |  |  | **M** |
| (Legakis et al., 2018) |  |  |  |  |  |  |  |  |  |  |  |  |  |  |  |  | **M** |
| (Lin et al., 2018) |  |  |  |  |  |  |  |  |  |  |  |  |  |  |  |  | **L** |
| (Nie et al., 2018) |  |  |  |  |  |  |  |  |  |  |  |  |  |  |  |  | **M** |
| (Vitet et al., 2018) |  |  |  |  |  |  |  |  |  |  |  |  |  |  |  |  | **M** |
| (Zhang et al., 2018) |  |  |  |  |  |  |  |  |  |  |  |  |  |  |  |  | **L** |

Abbreviations: M – Moderate; L – Low.

11 – Abstract; 12a and 12b – Background; 13 – Objectives; 14 – Ethical statement; 15 – Housing and Husbandry; 16a – 16c – Animal care and monitoring; 17a and 17b – Interpretation/Scientific implications; 18 – Generalisability/Translation; 19 – Protocol registration; 20 – Data Access; 21 – Declaration of interest.

**Supplementary Table 5 –** Summary of the ARRIVE Recommended Set.
