# Supplementary material for: Preclinical research in paclitaxel-induced neuropathic pain: a systematic review
Source: Front Vet Sci. 2023 Dec 18;10:1264668. doi: 10.3389/fvets.2023.1264668 (PMC10766764; doi:10.3389/fvets.2023.1264668)
Supplement: Supplementary file 6 [file Table_6.docx]

| **SYRCLE's Risk of Bias** | | | | | | | | | | |
| --- | --- | --- | --- | --- | --- | --- | --- | --- | --- | --- |
| **REF** | **Q1** | **Q2** | **Q3** | **Q4** | **Q5** | **Q6** | **Q7** | **Q8** | **Q9** | **Q10** |
| (Balkrishna et al., 2022) |  |  |  |  |  |  |  |  |  |  |
| (Cristiano et al., 2022) |  |  |  |  |  |  |  |  |  |  |
| (Ezaka et al., 2022) |  |  |  |  |  |  |  |  |  |  |
| (Karmakar et al., 2022) |  |  |  |  |  |  |  |  |  |  |
| (Li et al., 2022a) |  |  |  |  |  |  |  |  |  |  |
| (Lin et al., 2022) |  |  |  |  |  |  |  |  |  |  |
| (Ma et al., 2022) |  |  |  |  |  |  |  |  |  |  |
| (Nasser et al., 2022) |  |  |  |  |  |  |  |  |  |  |
| (Park et al., 2022) |  |  |  |  |  |  |  |  |  |  |
| (Paton et al., 2022) |  |  |  |  |  |  |  |  |  |  |
| (Sezer et al., 2022) |  |  |  |  |  |  |  |  |  |  |
| (Wang et al., 2022) |  |  |  |  |  |  |  |  |  |  |
| (Alkislar et al., 2021) |  |  |  |  |  |  |  |  |  |  |
| (Caillaud et al., 2021a) |  |  |  |  |  |  |  |  |  |  |
| (Caillaud et al., 2021b) |  |  |  |  |  |  |  |  |  |  |
| (Chen et al., 2021) |  |  |  |  |  |  |  |  |  |  |
| (Chou et al., 2021) |  |  |  |  |  |  |  |  |  |  |
| (Cuozzo et al., 2021) |  |  |  |  |  |  |  |  |  |  |
| (Foss et al., 2021) |  |  |  |  |  |  |  |  |  |  |
| (Garrido-Suárez et al., 2021) |  |  |  |  |  |  |  |  |  |  |
| (Ilari et al., 2021) |  |  |  |  |  |  |  |  |  |  |
| (Kim et al., 2021) |  |  |  |  |  |  |  |  |  |  |
| (Ma et al., 2021) |  |  |  |  |  |  |  |  |  |  |
| (Meregalli et al., 2021) |  |  |  |  |  |  |  |  |  |  |
| (Semis et al., 2021) |  |  |  |  |  |  |  |  |  |  |
| (Son et al., 2021) |  |  |  |  |  |  |  |  |  |  |
| (Takanashi et al., 2021) |  |  |  |  |  |  |  |  |  |  |
| (Wang et al., 2021a) |  |  |  |  |  |  |  |  |  |  |
| (Wang et al., 2021b) |  |  |  |  |  |  |  |  |  |  |
| (Zhang et al., 2021) |  |  |  |  |  |  |  |  |  |  |
| (Zhong et al., 2021) |  |  |  |  |  |  |  |  |  |  |
| (Balkrishna et al., 2020) |  |  |  |  |  |  |  |  |  |  |
| (Biggerstaff et al., 2020) |  |  |  |  |  |  |  |  |  |  |
| (Brewer et al., 2020) |  |  |  |  |  |  |  |  |  |  |
| (Chen et al., 2020) |  |  |  |  |  |  |  |  |  |  |
| (Costa-Pereira et al., 2020a) |  |  |  |  |  |  |  |  |  |  |
| (Costa-Pereira et al., 2020b) |  |  |  |  |  |  |  |  |  |  |
| (Ferrari et al., 2020) |  |  |  |  |  |  |  |  |  |  |
| (Hacimuftuoglu et al., 2020) |  |  |  |  |  |  |  |  |  |  |
| (Huang et al., 2020) |  |  |  |  |  |  |  |  |  |  |
| (Huynh et al., 2020) |  |  |  |  |  |  |  |  |  |  |
| (Kamata et al., 2020) |  |  |  |  |  |  |  |  |  |  |
| (Kim et al., 2020) |  |  |  |  |  |  |  |  |  |  |
| (Liang et al., 2020) |  |  |  |  |  |  |  |  |  |  |
| (Liu et al., 2020) |  |  |  |  |  |  |  |  |  |  |
| (Lu et al., 2020) |  |  |  |  |  |  |  |  |  |  |
| (Wang et al., 2020) |  |  |  |  |  |  |  |  |  |  |
| (Zhang et al., 2020) |  |  |  |  |  |  |  |  |  |  |
| (Zhao et al., 2020) |  |  |  |  |  |  |  |  |  |  |
| (Zhou et al., 2020a) |  |  |  |  |  |  |  |  |  |  |
| (Zhou et al., 2020b) |  |  |  |  |  |  |  |  |  |  |
| (Chen et al., 2019) |  |  |  |  |  |  |  |  |  |  |
| (Inyang et al., 2019) |  |  |  |  |  |  |  |  |  |  |
| (Kaur and Muthuraman, 2019) |  |  |  |  |  |  |  |  |  |  |
| (Li et al., 2019a) |  |  |  |  |  |  |  |  |  |  |
| (Li et al., 2019b) |  |  |  |  |  |  |  |  |  |  |
| (Mao et al., 2019) |  |  |  |  |  |  |  |  |  |  |
| (Ramakrishna et al., 2019) |  |  |  |  |  |  |  |  |  |  |
| (Sivanesan et al., 2019) |  |  |  |  |  |  |  |  |  |  |
| (Slivicki et al., 2019) |  |  |  |  |  |  |  |  |  |  |
| (Tonello et al., 2019) |  |  |  |  |  |  |  |  |  |  |
| (Wu et al., 2019a) |  |  |  |  |  |  |  |  |  |  |
| (Wu et al., 2019b) |  |  |  |  |  |  |  |  |  |  |
| (Al-Mazidi et al., 2018) |  |  |  |  |  |  |  |  |  |  |
| (Ba et al., 2018) |  |  |  |  |  |  |  |  |  |  |
| (Legakis et al., 2018) |  |  |  |  |  |  |  |  |  |  |
| (Lin et al., 2018) |  |  |  |  |  |  |  |  |  |  |
| (Nie et al., 2018) |  |  |  |  |  |  |  |  |  |  |
| (Vitet et al., 2018) |  |  |  |  |  |  |  |  |  |  |
| (Zhang et al., 2018) |  |  |  |  |  |  |  |  |  |  |

Q1 - Was the allocation sequence adequately generated and applied? Q2 - Were the groups similar at baseline or were they adjusted for confounders in the analysis?; Q3 - Was the allocation to the different groups adequately concealed during?; Q4 - Were the animals randomly housed during the experiment?; Q5 - Were the caregivers and/or investigators blinded from knowledge which intervention each animal received during experiment?; Q6 - Were animals selected at random for outcome assessment?; Q7 - Was the outcome assessor blinded?; Q8 - Were incomplete outcome data adequately addressed?; Q9 - Are reports of the study free of selective outcome reporting?; Q10 - Was the study apparently free of other problems that could result in high risk of bias?

**Supplementary Table 6 -** SYRCLE’s Risk of Bias assessment.
